# Pennsylvania State Dental Society

**Published:** 1891-02

**Authors:** 


(To be continued.)

---

### PENNSYLVANIA STATE DENTAL SOCIETY.

The Twenty-second Annual Meeting of the Pennsylvania State Dental Society met in the Minnequa House, Minnequa Springs, Bradford Co., Pa., on Tuesday, July 29, 1890.

President J. C. M. Hamilton in the chair, who called the meeting to order at 10.30 A.M. The morning was devoted to routine business.

The afternoon session was opened by the annual address of the president, Dr. Hamilton, who called attention to the importance of improving the moral tone of students, and if found deficient in the moral sense they should be dropped from the rolls.

He laid special stress upon a "Scientific Knowledge of Man's Origin." The law of heredity was explained and the necessity of close attention to its requirements was considered at length.

He alluded to anomalies in teeth and the fact that the American teeth are poorer does not warrant the wholesale removal as witnessed.

He urged the Society to make an effort to arouse an interest in their meetings and to endeavor to induce a large attendance.

After some commendatory remarks on President Hamilton's address, Dr. Alonzo P. Beale, of Philadelphia, read a paper on "Inter-Dental Splints."

(For Dr. Beale's paper, see page 81.)

The subject was illustrated by a number of colored drawings which were frequently referred to by the essayist.

After the reading of Dr. Beale's paper the president announced the subject open for discussion.

Dr. H. Leffman thought the paper demonstrated the necessity of physicians being more familiar with the different methods spoken of in the paper.

Dr. Beck, who has had much experience in this kind of work from practising in a mining region, spoke of special cases which had come under his observation necessitating the use of bands and wires, it being impossible to take impressions as Dr. Beale suggested, and, while not perfect when healed, the patients so treated all presented a very respectable appearance. He was well pleased with the paper, and would take advantage of the suggestions offered at the first opportunity which should present itself.

Dr. Roberts thought Dr. Beale had so thoroughly covered the subject that it left nothing for him to suggest.

Dr. W. H. Fundenberg has had considerable experience with maxillary fractures, but as the cases always come from physicians several days after the accident, much valuable time has necessarily been lost. He spoke of a case in the person of a little girl where the extent of the fracture was so great that no impression of the teeth could be obtained. An impression of the chin was taken, corresponding to which a cap splint was made, which was held in position until the parts were united.

Dr. Hamilton had a case the treatment of which, he said, was somewhat crude though effectual. The anterior portion of the maxilla had been smashed in. Treatment consisted simply in saturating a sponge with plaster of Paris, placing it in the mouth, which being closed upon, it was held in position by means of bandages for eight days, at the end of which time this improvised splint was renewed. Four weeks of this treatment resulted in a very satisfactory healing of the parts.

Dr. Boice thought that ingenuity and simplicity entered largely into the successful treatment of fractures. He never had any success with the correct articulation method Dr. Beale speaks of.

Dr. Beale explained that as the splints are stationary the measurements are intended mostly to approximate the position of the splints; that ligatures were very good for certain cases, but that splints restored more perfectly the articulation. He called especial attention to the covering of the casts with tin-foil for reasons given in the paper.

Dr. J. C. Green thought the society should feel very grateful to Dr. Beale for his excellent paper and especially thankful for the advantages we possess to-day over the time thirty years ago when we had no such material as vulcanizable rubber from which to make inter-dental splints.

Dr. Klump has made a number of splints on the principle suggested by the essayist, and they have given uniform satisfaction.

Subject passed.

